# Promoting Effect of L-Fucose on the Regeneration of Intestinal Stem Cells through AHR/IL-22 Pathway of Intestinal Lamina Propria Monocytes

**DOI:** 10.3390/nu14224789

**Published:** 2022-11-12

**Authors:** Chen Tan, Gaichao Hong, Zhe Wang, Caihan Duan, Lingzhi Hou, Junhao Wu, Wei Qian, Chaoqun Han, Xiaohua Hou

**Affiliations:** Division of Gastroenterology, Union Hospital, Tongji Medical College, Huazhong University of Science and Technology, Wuhan 430022, China

**Keywords:** L-fucose, intestinal stem cell, intestinal lamina propria monocytes, AHR, IL-22, organoids

## Abstract

The recovery of the intestinal epithelial barrier is the goal for curing various intestinal injurious diseases, especially IBD. However, there are limited therapeutics for restoring intestinal epithelial barrier function in IBD. The stemness of intestinal stem cells (ISCs) can differentiate into various mature intestinal epithelial cells, thus playing a key role in the rapid regeneration of the intestinal epithelium. IL-22 secreted by CD4^+^ T cells and ILC3 cells was reported to maintain the stemness of ISCs. Our previous study found that L-fucose significantly ameliorated DSS-induced colonic inflammation and intestinal epithelial injury. In this study, we discovered enhanced ISC regeneration and increased intestinal IL-22 secretion and its related transcription factor AHR in colitis mice after L-fucose treatment. Further studies showed that L-fucose promoted IL-22 release from CD4^+^ T cells and intestinal lamina propria monocytes (LPMCs) via activation of nuclear AHR. The coculture system of LPMCs and intestinal organoids demonstrated that L-fucose stimulated the proliferation of ISCs through an indirect manner of IL-22 from LPMCs via the IL-22R-p-STAT3 pathway, and restored TNF-α-induced organoid damage via IL-22-IL-22R signaling. These results revealed that L-fucose helped to heal the epithelial barrier by accelerating ISC proliferation, probably through the AHR/IL-22 pathway of LPMCs, which provides a novel therapy for IBD in the clinic.

## 1. Introduction

Inflammatory bowel disease (IBD) is a nonspecific autoimmune disorder with intestinal mucosal damage as the main pathological manifestation, mainly including ulcerative colitis and Crohn’s disease [1,2]. Although the pathological mechanism of IBD remains obscure, it is generally accepted that under the influence of environmental factors, genetically sensitive individuals develop intestinal immune dysregulation, which leads to the destruction of the intestinal epithelial barrier [3,4]. Repair of the intestinal epithelial barrier is the goal of the treatment of IBD. Conventional treatment for IBD lies in immunoregulation (e.g., 5-aminosalicylic acid), immunosuppressants (e.g., glucocorticoids), biological therapy (e.g., antitumor necrosis factor-α monoclonal antibody), intestinal flora regulation (e.g., fecal microbiota transplantation), etc. [5,6,7]. However, the present therapeutic drugs for IBD focus on controlling inflammation with limited effects in restoring intestinal epithelial barrier function, highlighting an unmet need for new treatment to cure IBD.

Intestinal epithelial cells (IECs) are derived from intestinal stem cells (ISCs), which are the driving factors for the stability and regeneration of the intraepithelial environment [8,9]. Recent studies revealed that the immune system could maintain ISC stemness, thus promoting the regeneration of the intestinal epithelium. For example, Zhu et al. proved that IL-13 produced by group 2 innate lymphoid cells in the crypt niche bonds to IL-13Rα1 on ISCs and activates signaling mediated by IL-13-IL-13R, resulting in the activation of the β-catenin pathway and the proliferation of Lgr5^+^ ISCs [10]. Moshe Biton et al. found that intestinal organoids stimulated by Th cytokines lead to Lgr5^+^ ISC renewal and differentiation in the opposite way: proinflammatory signals promote differentiation, while regulatory cells and cytokines reduce differentiation [11]. Alan M Hanash et al. reported that innate lymphoid cells (ILCs), efficient producers of interleukin-22 (IL-22) after intestinal injury, promoted the growth of mouse intestinal organoids in an IL-22-dependent fashion [12].

L-fucose is a dietary additive, also known as 6-deoxy-L-galactose, commonly found in natural plants, including brown algae and tragacanth gum [13,14]. As a dietary sugar, L-fucose was reported to show a good ability to improve intestinal inflammation in mice [15]. Our previous study found that the exogenous administration of L-fucose effectively alleviated DSS-induced colitis in mice, including chronic and acute colitis, and it works by relieving intestinal inflammation and restoring the intestinal epithelial barrier [16,17,18]. In addition, we found for the first time that L-fucose significantly increased the number of ISCs in colitis mice. Therefore, it is speculated that L-fucose mediates repair after intestinal epithelial barrier injury through ISC renewal. However, whether L-fucose acts on ISCs in a direct or indirect way remains obscure.

In this study, we aimed to investigate the stimulatory effect and specific mechanism of L-fucose on ISCs in colitis mice. Intestinal IL-22 and ISC proliferation were detected in the mouse colon. The CD4^+^ T cells and LPMCs were extracted and treated with L-fucose to observe its effect on IL-22 secretion. Furthermore, we constructed the in vitro models of intestinal organoids cultured alone and the coculture system of LPMCs with intestinal organoids, which were treated with L-fucose to explore its direct or indirect influence on ISCs.

## 2. Materials and Methods

### 2.1. Animals

Male C57BL/6 J mice (6–8 weeks old, 21 ± 2 g) were purchased from Beijing Huafukang Biotechnology Co., Ltd. (Beijing, China). Animals were kept under specific pathogen-free conditions at room temperature of 22 ± 2 °C and relative humidity of 55 ± 5% on a 12 h light/dark cycle. All mice were given adaptive feeding for at least one week before formal experimentation. Water and feed were provided freely throughout the experiment. This experiment was approved by the Animal Care and Utilization Committee of Tongji Medical College, Huazhong University of Science and Technology (approval number: 2529).

### 2.2. Induction of Experimental Colitis and Animal Treatment

Mice were randomly assigned to four groups (*n* = 20/group): (1) the control group (Con), (2) the L-fucose-fed group (FUC), (3) the DSS-fed group (DSS) and (4) the DSS- and L-fucose-fed group (DSS + FUC). A chronic colitis mouse model induced by DSS was established according to a previously described method with minor modification [16]. In brief, 2% DSS (molecular weight 36–50 kDa; MP Biomedicals, Santa Ana, CA, USA) was dissolved in drinking water, and mice were given free access to 2% DSS for 7 days and then washed out for 14 days with normal drinking water. The above procedure was repeated for three cycles. The gavage of L-fucose (250 mg/kg, Sigma-Aldrich, St. Louis, MO, USA) started 3 days before the experiment and conducted daily until the end of the experiment. Meanwhile, the same volume and frequency of water was administered in the control group and DSS group. During the experiment, body weight, stool consistency and hematochezia status were monitored every other day to score the disease activity index (DAI). The DAI score was defined as the summation of weight loss index (0–4), the stool consistency index (0–4), and fecal bleeding index (0–4) [19]. On day 63, the mice were sacrificed and the length of the colons was measured. For histological analysis, the distal colon specimens were fixed in the 4% paraformaldehyde for 24 h, embedded in paraffin, sliced, and then stained with hematoxylin and eosin (H&E). The histological analysis of the colon was performed as previously described [20]. The remaining colon tissues were frozen under liquid nitrogen and stored at −80 °C for further treatment.

### 2.3. Crypt Isolation and Intestinal Organoid Culture

Intestinal organoids were obtained from the intestines of 8-week-old C57BL/6 mice as previously described [21]. Briefly, small intestines were dissected and cut into pieces and then washed with DPBS for twenty times until the supernatant became clear. Afterwards, the pieces were incubated in DPBS with 2.5 mM EDTA for 30 min on ice. Subsequently, the intestine piece mixture was shaken vigorously and passed through a 70 μm cell strainer, and crypt fractions were isolated via centrifugation at 290× *g* for 5 min. To remove single cells, the obtained crude crypts were purified by centrifugation at 200× *g* for 3 min. Then, the crypts were mixed with equal volumes of Matrigel (BD Bioscience/Corning, New York, NY, USA) and IntestiCult™ Organoid Growth Medium (Stemcell Technologies, Vancouver, BC, Canada) and seeded in 24-well plates. Put the culture plate into an incubator at 37 °C in a 5% CO_2_ atmosphere for 15 min. The IntestiCult™ Organoid Growth Medium was added after Matrigel solidification. The detailed methods of organoid passaging are described as follows. First, the medium was aspirated before adding 1 mL of cold PBS per well and the plate was placed on ice to thaw the medium. The cells were pipetted up and down until no solid pieces of Matrigel remained. The organoids were spun down at 290× *g* for 5 min at 4 °C and the PBS was removed. Subsequently, organoids were washed with DMEM/F12 (HyClone, Logan, UT, USA), purified by centrifugation at 200× *g* for 3 min, and resuspended in an equal volume of Matrigel/medium. Finally, 50 μL of organoid mixture was planted per well in a 24-well plate and incubated at 37 °C until the Matrigel polymerized before covering with IntestiCult™ Organoid Growth Medium.

### 2.4. Preparation of CD4^+^ T Cells from Mouse Spleen

The CD4^+^ T cells were sorted from fresh spleens of male C57BL6/J mice aged 6–8 weeks using a MojoSort™ mouse CD4^+^ T-cell isolation kit (BioLegend, San Diego, CA, USA) according to the manufacturer’s protocol. The isolated CD4^+^ T cells were cultured in 1640-cell culture medium supplemented with 1% streptavidin, 1% L-glutamine solution and 10% fetal bovine serum. For the in vitro experiment, cells were stimulated with 5 μg/mL anti-mouse CD3 antibody (BE0002, Bio X Cell, Lebanon, NH, USA ) and 2 μg/mL anti-mouse CD28 antibody (BE0328, Bio X Cell) and then divided into the control, L-fucose (5 mg/mL) and L-fucose (5 mg/mL) +CH223191 (3 μM) (C8124, Sigma-Aldrich, St. Louis, MO, USA) groups. After 5 days of intervention at 37 °C and 5% CO_2_, the culture supernatant was collected, and RNA, total protein and nuclear protein were extracted from CD4^+^ T cells as described in Section 2.12 and Section 2.13.

### 2.5. Preparation of Intestinal Lamina Propria Monocytes (LPMCs)

The small intestinal lamina propria monocytes (LPMCs) were harvested according to a previous study with a slight modification [12]. Briefly, small intestines were cut lengthwise, washed with ice-cold PBS and cut into large pieces. Then, the pieces were placed in 40 mL of epithelial cell dissociation solution [Ca2^+^ and Mg2^+^-free PBS supplemented with 2.5 mM EDTA (Solarbio, Peking, China), 10 mM HEPES buffer (Sigma-Aldrich, St. Louis, MO, USA) and 1 mM DTT buffer (Sigma-Aldrich, St. Louis, MO, USA)] at 37 °C and shaken at 150 rpm for 20 min. Subsequently, the fragments were cut into small pieces and digested in lamina propria digestive solution [RPMI1640 (Gibco, Life Technologies, New York, NY, USA), supplemented with 1 mg/mL Collagenase IV (Roche, BSL), 1 U/mL DNase I (Roche) and 1% penicillin/streptomycin (Gibco, Life Technologies, Carlsbad, CA, USA)] at 37 °C and shaken at 150 rpm for 15 min. Afterwards, the supernatant was filtered with a 70 μm cell strainer, and the filtrate was centrifuged at 150 rpm for 5 min and washed with RPMI medium supplemented with 5% FBS (Gibco, Life Technologies, Carlsbad, CA, USA). After centrifugation, the cells were resuspended in 40% Percoll solution (in PBS), overlaid on 80% Percoll solution, and then centrifuged at 500× *g* for 20 min at 20 °C. After spinning, the interface containing the LPMCs was aspirated and washed in medium for subsequent experiments. For in vitro experiments, LPMCs were divided into control, L-fucose (5 mg/mL) and L-fucose (5 mg/mL) +CH223191 (3 μM) groups as before and cultured in RPMI 1640 medium supplemented with 10% FBS and 1% penicillin/streptomycin for 24 h, and a leukocyte activation cocktail (BD Biosciences, New York, NY, USA) was added to the medium in the last 6 h.

### 2.6. Coculture System of LPMCs and Organoids

For coculture experiments, the isolated LPMCs were cultured with intestinal crypts at a ratio of 20:1 in Matrigel according to previous work, though with a slight modification [12]. The effective concentration of L-fucose on ISC proliferation was selected in the concentration gradient experiment (Figure S1). L-fucose (5 mg/mL) and murine anti-IL-22 neutralizing antibody (0.1 μg/mL, AF582, R&D system, Minneapolis, MN, USA) were added independently or simultaneously to the culture medium for 5 days, and the medium was changed every two days. Murine TNF-α (60 ng/mL, Peprotech, Rocky Hill, NJ, USA) was used to induce the inflammatory model for 24 h.

### 2.7. Flow Cytometry Analysis

After the intervention, the CD4^+^ T cells and intestinal LPMCs were stained with fluorescent mouse antibodies specific for AF-700-CD45 (1:200, BD Biosciences, San Jose, CA, USA) and FITC-CD4 (1:200, BD Biosciences, San Jose, CA, USA) at 4 °C for 30 min. Then, the cells were fixed and permeabilized with fixation/permeabilization solution (BD Biosciences, San Jose, CA, USA) for 30 min and washed with PBS. Next, LPMCs were stained with PE-IL-22 antibody (1:100, eBioscience, San Diego, CA, USA) and BV421-ROR-γt antibody (1:100, BD Biosciences, San Jose, CA, USA), and the CD4^+^ T cells were stained with PE-IL-22 antibody (1:100, eBioscience, San Diego, CA, USA) at 4 °C for 30 min. After washing three times with PBS, the cells were analyzed by FACS (BD Pharmingen, San Diego, CA, USA). Data were analyzed by FlowJo V10 software (Tree Star, Ashland, OR, USA).

### 2.8. Intestinal Organoids Measurement

The measurement of organoids was performed according to a previous description [22]. Briefly, several random nonoverlapping fields were selected from each well under light microscopy. After setting the scale bar, the area of organoids was manually measured by ImageJ software (V1.8.0, NIH, Bethesda, MD, USA). Organoids touching the edges of the image were excluded from the count. In addition, the total number of organoids per graph and the number of organoid buds were manually counted to assess growth efficiency.

### 2.9. EdU Staining

Cell proliferation in organoids was assessed by the BeyoClick™ EdU Cell Proliferation Kit with Alexa Fluor 594 (Ca.No: C0078S, Beyotime Biotechnology, Shanghai, China) according to the manufacturer’s instructions. Briefly, the organoids were treated with 10 μM of EdU at 37 °C for 2 h and then fixed in 4% paraformaldehyde at room temperature for 15 min. After permeabilization with 0.3% Triton X-100, the organoids were reacted with reaction mixture for 30 min. Subsequently, the nuclei were stained with DAPI for 15 min. The EdU-stained 3D organoids were visualized under a Zeiss 710 Laser Scanning confocal microscope (CarlZeiss, Oberkochen, Germany). The numbers of EdU^+^ cells in per organoid were analyzed by ImageJ software.

### 2.10. Immunohistochemistry (IHC) and Immunofluorescent (IF) Assay

For immunohistochemical staining of Ki67, the terminal colon specimens were fixed with paraformaldehyde, embedded in paraffin and sectioned as described previously [23]. First, the slides were dried and dewaxed with xylene for two cycles and then heated with citrate buffer solution for antigen retrieval. After cooling, the slides were blocked with 10% donkey serum at room temperature for 1 h and then incubated with the anti-mouse Ki67 antibody (1:200, ab15580, Abcam) at 4 °C overnight. The next day, the slides were washed with PBS and incubated with ready-to-use IHC detection reagents (Cell Signaling, Boston, MA, USA) at room temperature for 1 h. A DAB kit (Cell Signaling, Boston, MA, USA) was used to detect the signal. The slides were viewed and recorded on an Olympus BX51 microscope (Olympus, Tokyo, Japan).

For immunofluorescent staining, after the same deparaffinization and antigen retrieval steps mentioned above, the slides were permeabilized with 0.3% Triton X-100 at room temperature for 20 min, followed by blocking with 10% donkey serum for 1 h. Then, the sections were incubated with anti-mouse Lgr5 antibody (1:200, A10545, ABclonal, Woburn, MA, USA) and anti-mouse IL-22R antibody (1:50, MAB42941, R&D system) at 4 °C overnight. For pSTAT3 staining of organoids, the anti-mouse pSTAT3 antibody (1:200, 4113, Cell Signaling, Boston, MA, USA) was used as the primary antibody. The next day, the samples were then incubated with donkey anti-rabbit Alexa Fluor 488 (1:200, ANT024S, AntGene, Wuhan, China), donkey anti-mouse Alexa Fluor 488 (1:200, ANT023S, AntGene) or donkey anti-rat Alexa Fluor 594 (1:200, ANT036S, AntGene) at room temperature for 1 h, followed by incubation with an anti-fluorescence quencher with DAPI (ANT063, AntGene). Fluorescence images were viewed and collected under a Zeiss 710 Laser Scanning confocal microscope.

### 2.11. Cytokine Detection

After treatment of the coculture system of LPMCs and organoids, the culture medium was collected and stored at −20 °C for ELISA analysis of TNF-α. Colonic specimens were collected from euthanized mice, homogenized and centrifuged, and the supernatant was stored at −20 °C for ELISA analysis of IL-22. A Mouse Interleukin 22 (IL-22) ELISA Kit (MU30582, Bio-Swamp, Wuhan, China) and Mouse Tumor necrosis factor α ELISA Kit (MU30030, Bio-Swamp) were utilized to measure the protein concentrations of IL-22 and TNF-α proteins level according to the manufacturer’s instructions. The absorbance was obtained with a microplate reader (Biotek Instruments, Inc., Winooski, VT, USA) at the corresponding nanometer wavelength.

### 2.12. Quantitative RT-PCR

Both colonic tissues and intestinal organoids were harvested after treatment. Briefly, total RNA was isolated with TRIzol (TaKaRa, Dalian, China). Complementary DNAs (cDNAs) were obtained by reverse transcription using PrimeScript™ RT Master Mix (TaKaRa, Dalian, China). Gene expression was detected through amplification of cDNA fragments with SYBR Premix Ex TaqTM (TaKaRa) on a Roche LightCycler R480 (Roche, Switzerland). The relative expression levels of genes were normalized to the housekeeping gene GAPDH. All mRNA primer sequences are listed in Supplementary Table S1.

### 2.13. Western Blotting

Western blotting analysis was performed according to a previous description [17]. Briefly, total proteins were collected from tissues and organoids with RIPA lysis buffer (Beyotime, Shanghai, China) supplemented with 1% (*v*/*v*) phenylmethyl sulfonyl fluoride (PMSF), 1% (*v*/*v*) protease inhibitor and 1% (*v*/*v*) phosphatase inhibitor. Nuclear protein extraction from colon tissues and lymphocytes was performed according to the manufacturer’s instructions of the nuclear protein extraction kit (Solarbio, Peking, China). The bicinchoninic acid (BCA) assay kit (Thermo Fisher Scientific, Waltham, MA, USA) was used to detect protein concentrations. Equal amounts of denatured protein samples were separated by sodium dodecyl sulfate-polyacrylamide gel electrophoresis (SDS-PAGE) and then electrophoretically transferred to PVDF membranes (Millipore Corp., Burlington, MA, USA). After blocking with 5% skimmed milk in TBS buffer containing 0.1% Tween-20, the membranes were incubated with rabbit anti-Lgr5 (1:1000, A10545, ABclonal), rabbit anti-IL-22 (1:1000, A6216, ABclonal), rabbit anti-β-catenin (1:1000, 9582, Cell Signaling), rabbit anti-STAT3 (1:1000, A19566, ABclonal), rabbit anti-phospho-STAT3-Y705 (1:1000, 4113, Cell Signaling), rabbit anti-AHR (1:1000, A00225-3, Boster, Wuhan, China) and rabbit anti-CYP1A1 (1:1000, A2159, ABclonal) antibodies. The mouse anti-β-actin (1:3000, 66009-1-Ig, Proteintech, Chicago, IL, USA) and rabbit anti-Lamin b1 (1:1000, YT5180, ImmunoWay, CA, USA) antibodies were used for normalization. Afterwards, the membranes were incubated with goat anti-rabbit secondary antibodies (1:3000, PR30011, Proteintech) and goat anti-mouse secondary antibodies (1:3000, PR30012, Proteintech). The protein bands were visualized using an enhanced chemiluminescence (ECL) kit (Beyotime, Shanghai, China) under an Image Reader LAS-4000 imaging system (FUJIFLIM, Tokyo, Japan).

### 2.14. Statistical Analysis

All data are reported as the mean ± standard deviation (mean ± SD). Two groups were analyzed by unpaired two-tailed Student’s *t* test; one-way ANOVA was employed to determine significant differences among multiple groups. *p* < 0.05 was considered statistically significant. SPSS 22.0 (IBM, Chicago, IL, USA) and Graphpad Prism software (8.0, GraphPad Software, San Diego, CA, USA) were used for data analysis.

## 3. Results

### 3.1. L-Fucose Ameliorates DSS-Induced Chronic Colitis

**Figure 1:**
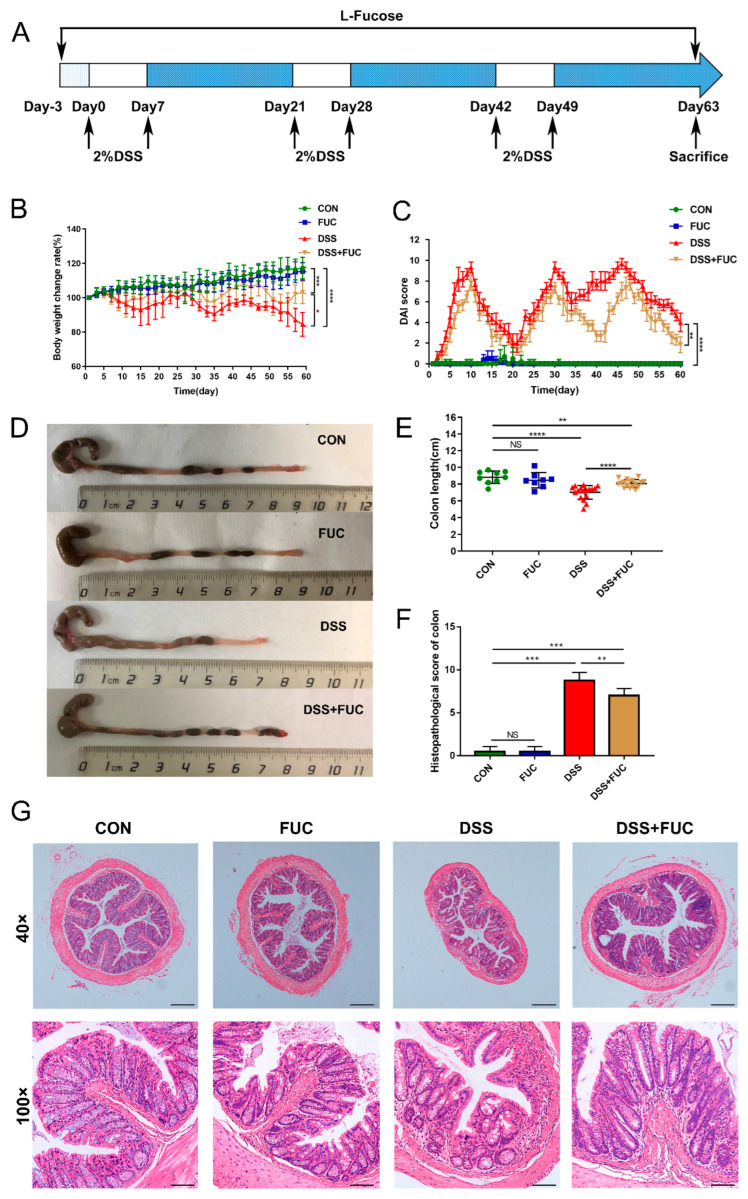
**Amelioration of DSS-induced chronic colitis with L-fucose treatment.** (**A**) Schematic diagram illustrating the experimental design. (**B**) Body weight percentage changes in each group. (**C**) Disease activity index (DAI) evaluations. (**D**) Comparisons of colon length in each group. (**E**) Quantitative analysis of colon length. (**F**) Histopathological scores of colons from each group of mice. (**G**) Representative images of H&E staining of colon sections. Magnification, 40× (above), 100× (below). Data are presented as the mean ± SD. * *p* < 0.05, ** *p* < 0.01, *** *p* < 0.001, **** *p* < 0.0001; NS, no significance.

To study the therapeutic effect of L-fucose on UC, a DSS-induced chronic colitis mouse model was established (Figure 1A). As shown in Figure 1B,C, the chronic colitis mice raised in a conventional way experienced periodic body weight loss and severe loose or bloody stools, as indicated by the DAI score. Compared with the mice in the DSS group, L-fucose significantly improved DSS-induced colitis with less weight loss (*p* = 0.0161) and a reduced DAI score (*p* = 0.0011). Colon shortening is one of the main characteristics of DSS-induced colitis (Figure 1D,E). The administration of L-fucose significantly reversed the colon shortening compared to their DSS-induced littermates (*p* < 0.0001). In addition, the histopathological analysis showed a consistent trend. Specifically, in the DSS group, obvious structural disorder, epithelial damage, mucosal ulceration, gland swelling, and inflammatory cell infiltration were observed in colon sections compared to the control and FUC groups (Figure 1F,G). Significantly reduced histological damage was observed in L-fucose-treated mice compared to the DSS group (*p* = 0.0319). These results demonstrate that L-fucose effectively ameliorates intestinal injury in DSS-induced colitis mice. However, it had no significant effect on the body weight, colon length, or intestinal mucosal structure of normal mice.

### 3.2. L-Fucose Stimulates ISC Regeneration and Intestinal IL-22 Secretion

**Figure 2:**
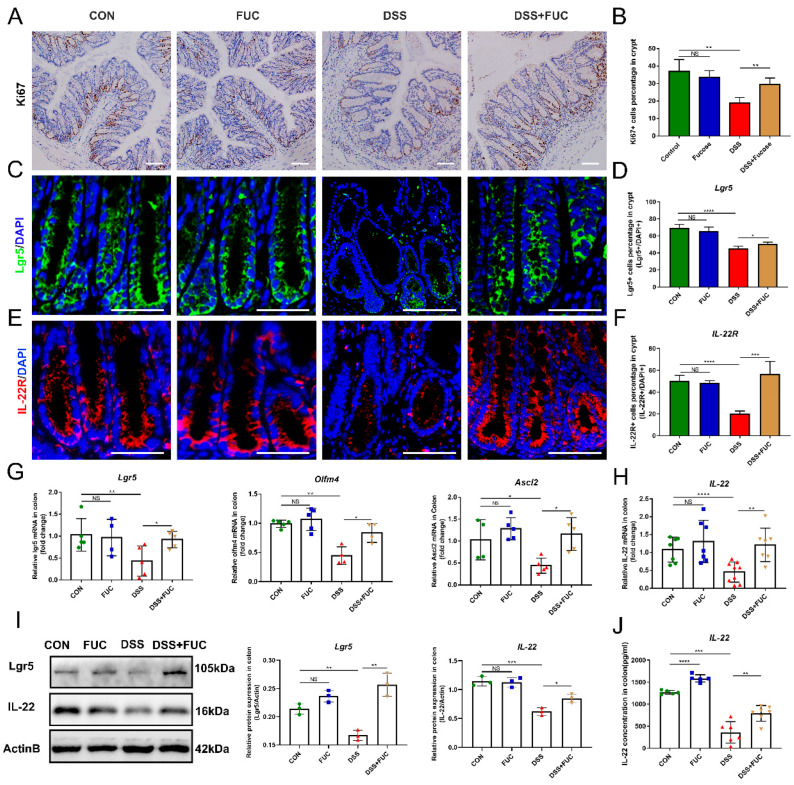
**Increased ISC regeneration and IL-22 secretion in mice treated with L-fucose.** (**A**) Representative images of Ki67 immunohistochemistry of the colon (scale bar, 100 μm). (**B**) Ki67^+^ cell percentage in crypts. (**C**) Representative images of Lgr5 (green) in the colon (scale bar, 50 μm). (**D**) Lgr5-positive cell percentage in crypts. (**E**) Representative images of IL-22R (red) in the colon (scale bar, 50 μm). (**F**) IL-22R-positive cell percentage in crypts. Quantitative reverse-transcriptase polymerase chain reaction (qRT-PCR) analysis of (**G**) Lgr5, Olfm4, Ascl2 and (**H**) IL-22 in the colon. (**I**) Western blotting analysis of Lgr5 and IL-22 proteins in the colon. (**J**) ELISA analysis of IL-22 in the colon. The scale bar represents 100 μm. Data are expressed as the mean ± SD. * *p* < 0.05, ** *p* < 0.01, *** *p* < 0.001, **** *p* < 0.0001; NS, no significance.

ISCs are critical for injury-induced intestinal regeneration. In this study, we assessed the effect of L-fucose on crypt proliferation in DSS-induced colitis mice by Ki67 immunohistochemical staining (Figure 2A). The reduction in Ki67 staining was more pronounced in the colon of DSS-treated mice than in normal and L-fucose-treated mice. Quantitative analysis showed that the Ki67-positive cell percentage was significantly restored in colitis mice after L-fucose administration (Figure 2B). Similarly, immunofluorescence for the ISC marker Lgr5 revealed that L-fucose significantly promoted the ISC regeneration in DSS-induced colitis compared to the DSS group (*p* = 0.0275) (Figure 2C,D). Likewise, real-time quantitative PCR showed that the mRNA expression of the ISC marker genes Lgr5, Olfm4 and Ascl2 was increased in the L-fucose-treated colitis mice (Figure 2G). In addition, we examined the secretion of IL-22 in colon tissue by Western blotting and ELISA analysis as well as the expression of IL-22R at the bottom of the crypt through immunofluorescence. As shown in Figure 2E,F, L-fucose significantly increased the number of IL-22R-positive cells compared to the DSS group (*p* = 0.0009). The protein and mRNA levels of IL-22 in the colon were observed to be increased after L-fucose administration in DSS-induced mice (Figure 2H–J). These data indicate that L-fucose can stimulate ISC regeneration after injury and promote intestinal IL-22 secretion and IL-22R expression at the crypt bottom. However, L-fucose has no significant effect on ISC proliferation or IL-22 in normal mice.

### 3.3. L-Fucose Activates the IL-22-Related Transcription Factor AHR in Mice

**Figure 3:**
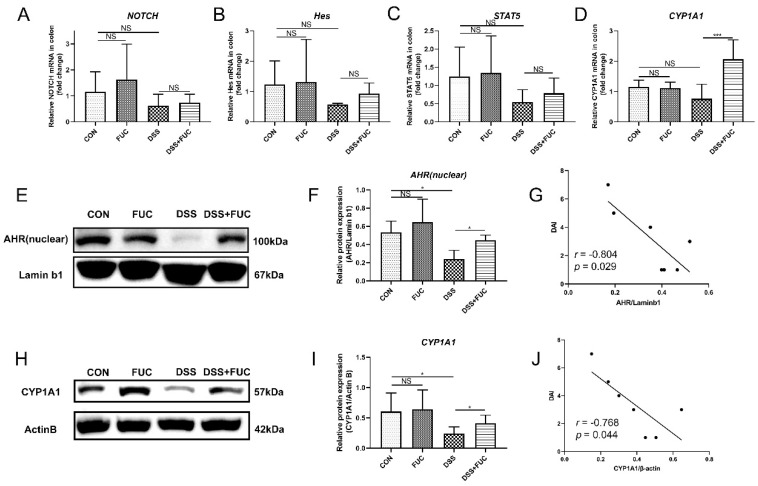
**High expression of IL-22-related transcription factors in the colon after L-fucose treatment.** qRT-PCR analysis of (**A**) NOTCH, (**B**) Hes, (**C**) STAT5 and (**D**) CYP1A1 in the colon. (**E**) Western blotting analysis of AHR protein in the colon nucleus. (**F**) Quantitative analysis of AHR protein in the colon nucleus. (**G**) Correlation analysis between DAI score and AHR protein. (**H**) Western blotting analysis of CYP1A1 protein in the colon. (**I**) Quantitative analysis of CYP1A1 protein in the colon. (**J**) Correlation analysis between DAI score and CYP1A1 protein. Data are expressed as the mean ± SD. * *p* < 0.05, *** *p* < 0.001; NS, no significance.

To explore how L-fucose regulates intestinal IL-22 secretion, we screened IL-22-related transcription factors via qRT-PCR analysis and Western blotting. Among the four important transcription factors, Notch, Hes and STAT5 showed no significant difference between the DSS and DSS + FUC groups (Figure 3A–C). While AHR and its target gene CYP1A1 were increased after L-fucose administration in DSS-induced colitis (Figure 3D–F,H,I). Furthermore, the expression of AHR and CYP1A1 proteins was significantly reduced in the DSS group compared to normal mice and showed a significant negative correlation with the DAI score (Figure 3G,J). These results suggest that L-fucose may promote intestinal IL-22 secretion by activating AHR expression.

### 3.4. L-Fucose Promotes the Secretion of IL-22 from CD4^+^ T Lymphocytes via AHR In Vitro

**Figure 4:**
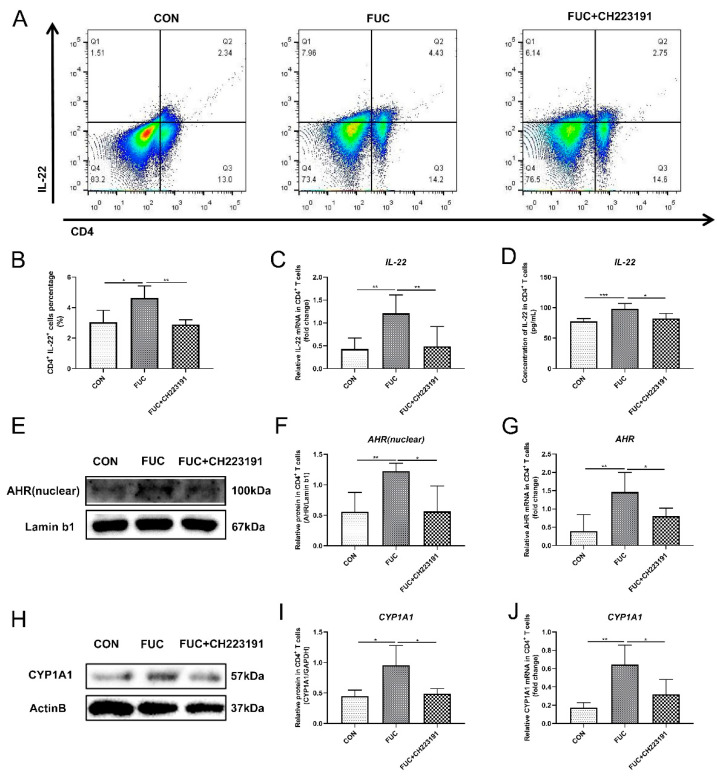
**Increased IL-22 secretion from CD4^+^ T lymphocytes through AHR activation after L-fucose treatment.** (**A**) Representative flow cytometric profiles of IL-22 secreting CD4^+^ cells. (**B**) Percentage of IL-22^+^ cells in CD4^+^ T cells. (**C**) qRT-PCR analysis of IL-22 in CD4^+^ T cells. (**D**) ELISA analysis of IL-22 in the culture supernatant of CD4^+^ T cells. (**E**) Western blotting analysis of AHR protein in CD4^+^ T-cell cell nuclei. (**F**) Quantitative analysis of AHR protein in CD4^+^ T-cell cell nuclei. (**G**) qRT-PCR analysis of AHR in CD4^+^ T cells. (**H**) Western blotting analysis of CYP1A1 in CD4^+^ T cells. (**I**) Quantitative analysis of CYP1A1 protein in CD4^+^ T cells. (**J**) qRT-PCR analysis of CYP1A1 in CD4^+^ T cells. Data are expressed as the mean ± SD. * *p* < 0.05, ** *p* < 0.01, *** *p* < 0.001; NS, no significance.

Since IL-22 in the mouse gut is partly derived from CD4^+^ T cells, we further investigated the specific mechanism by which L-fucose mediated intestinal IL-22 secretion in in vitro experiments of L-fucose intervention in CD4^+^ T lymphocytes, in the presence and absence of an AHR inhibitor (CH223191). The results of flow cytometry showed that L-fucose significantly increased the number of IL-22 positive cells in CD4^+^ T cells (*p* = 0.0309), while CH223191 inhibited this function of L-fucose (*p* = 0.007) (Figure 4A,B). In addition, both the mRNA and protein levels of IL-22 in CD4^+^ T cells were obviously elevated after L-fucose treatment, and this phenomenon was inhibited by CH223191 (Figure 4C,D). The above results preliminarily indicate that L-fucose may promote the secretion of IL-22 from CD4+ T cells through AHR. AHR exists in the cytoplasm, and when the ligand binds to AHR, the AHR-ligand complex is translocated from the cytoplasm to the nucleus, promoting the expression of downstream genes, including IL-22. Western blotting analysis showed that AHR in the nucleus of CD4^+^ T cells was significantly increased under the intervention of L-fucose (*p* = 0.0061), while CH223191 suppressed the nuclear expression of AHR (*p* = 0.0196) (Figure 4E,F). qRT-PCR analysis showed similar trends in AHR expression in CD4^+^ T cells (Figure 4G). Furthermore, compared to the control group, the AHR target protein CYP1A1 was significantly increased in CD4^+^ T cells in the L-fucose group (*p* = 0.0233) (Figure 4H,I). CH223191 significantly inhibited the expression of CYP1A1 protein in L-fucose-stimulated CD4^+^ T cells (*p* = 0.0299). The transcription level of CYP1A1 in CD4^+^ T cells among the three groups showed similar trends (Figure 4J). Based on the above results, L-fucose promotes the secretion of IL-22 from CD4^+^ T cells by upregulating the transcription factor AHR.

### 3.5. L-Fucose Promotes the Secretion of IL-22 from ILC3s via AHR In Vitro

**Figure 5:**
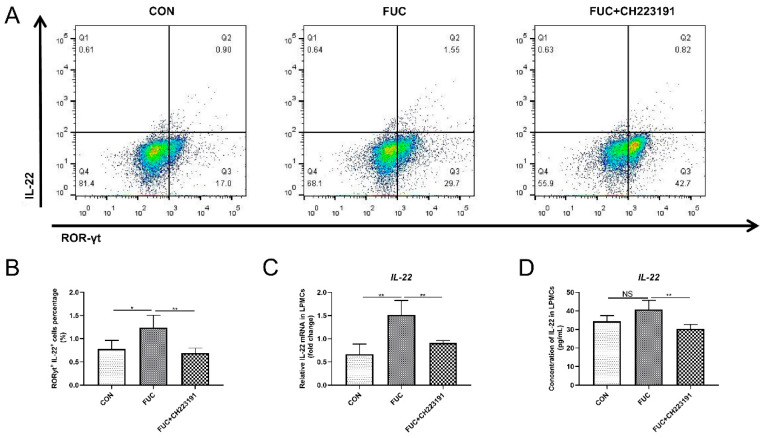
**Increased IL-22 release from ILC3 cells through AHR activation after L-fucose treatment.** (**A**) Representative flow cytometric profiles of IL-22^+^ ROR-γt^+^ cells in LPMCs. (**B**) Percentage of IL-22^+^ ROR-γt^+^ cells in LPMCs. (**C**) qRT-PCR analysis of IL-22 mRNA in LPMCs. (**D**) ELISA analysis of IL-22 in culture supernatant of LPMCs. Data are expressed as the mean ± SD. * *p* < 0.05, ** *p* < 0.01; NS, no significance.

In addition to being secreted by CD4^+^ T cells, intestinal IL-22 is also largely derived from the innate immune cell ILC3 [24]. ILC3s are widely distributed in intestinal lamina propria immune cells. To further explore whether L-fucose promotes IL-22 secretion from ILC3s via AHR activation, we isolated mouse intestinal LPMCs, treated them as described above and analyzed the proportion of ILC3s by flow cytometry. As shown in Figure 5A,B, compared to the control group, the proportion of IL-22^+^ROR-γt^+^ cells was significantly increased after L-fucose intervention (*p* = 0.0295). In contrast, CH223191 inhibited the IL-22-promotion effect of L-fucose (*p* = 0.0084). In addition, qRT-PCR analysis of IL-22 mRNA in LPMCs and ELISA assay of IL-22 in culture supernatant of LPMCs showed similar trends (Figure 5C,D). These data suggest that L-fucose may mediate the secretion of IL-22 from ILC3s through AHR activation.

### 3.6. L-Fucose Accelerates ISC Proliferation through IL-22 Secretion from LPMCs

**Figure 6:**
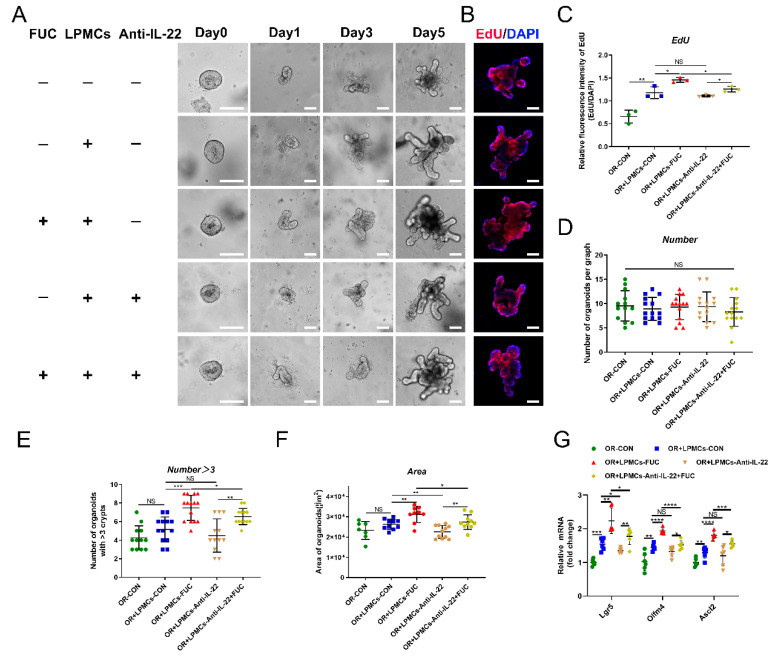
**Enhanced ISC proliferation via IL-22 from LPMCs after L-fucose treatment.** (**A**) Representative images of LPMC and intestinal organoid coculture experiments (scale bar, 100 μm). (**B**) EdU staining of organoids (scale bar, 100 μm). (**C**) Quantitative analysis of EdU fluorescence intensity. (**D**) Number of organoids per field of view. (**E**) Number of organoids with more than 3 buds per graph. (**F**) Average area of organoids per graph. (**G**) qRT-PCR analysis of Lgr5, Olfm4 and Ascl2 in organoids. Data are expressed as the mean ± SD. * *p* < 0.05, ** *p* < 0.01, *** *p* < 0.001, **** *p* < 0.0001; NS, no significance. +, with. −, without.

In the above experiments, we found that L-fucose promoted the proliferation of ISCs and the secretion of intestinal IL-22 after DSS damage. To investigate whether L-fucose accelerates ISC proliferation through IL-22, we performed LPMC—intestinal organoid coculture experiments. After the continuous administration of L-fucose for 5 days, the area and budding rate of organoids increased significantly in the presence of LPMCs. However, the anti-IL-22 antibody inhibited the promotion effect of L-fucose (Figure 6A,E,F). Figure 6B,C shows the proliferation rate of organoids by EdU staining. It is worth noting that the area of organoids in the coculture group was larger than that of the organoids cultured alone, and the proliferation rate was faster, which may be attributed to the IL-22 secreted by LPMCs in the basal state. In addition, no obvious growth-promoting effect was observed in the experiment of L-fucose directly intervening in organoids (Figure S2). To exclude the effect of organoid density on growth status, there was no significant difference in the number of organoids in random fields selected from each group (Figure 6D). The mRNA levels of Lgr5, Olfm4 and Ascl2 were also increased by L-fucose treatment in organoids and were reduced by anti-IL-22 antibody intervention (Figure 6G). These data indicate that L-fucose accelerates ISC proliferation through an indirect pathway of IL-22 from LPMCs, thereby rapidly restoring intestinal epithelial damage.

### 3.7. L-Fucose Stimulates ISC Proliferation through IL-22 from LPMCs via the IL-22R-p-STAT3 Pathway

**Figure 7:**
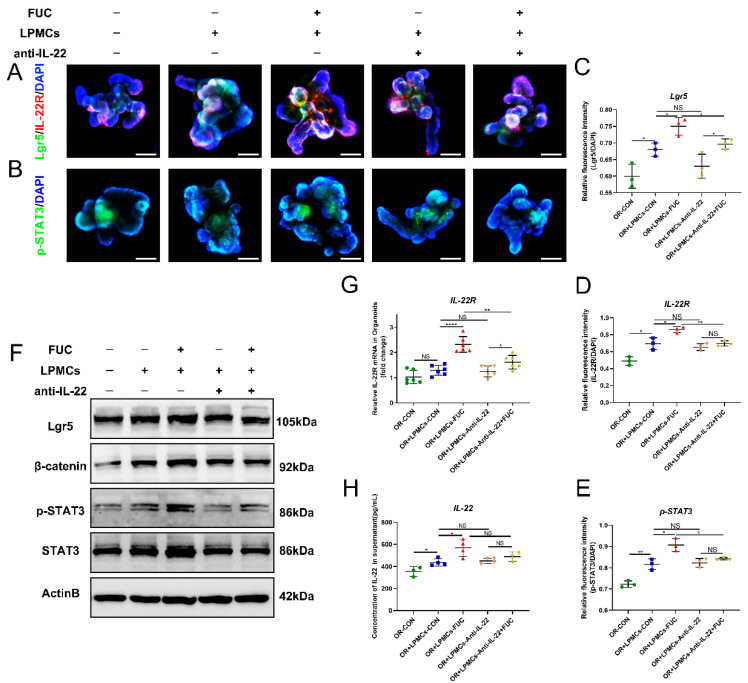
**IL-22R-p-STAT3 pathway mediated by IL-22 secretion of LPMCs in ISC proliferation after L-Fucose treatment.** Representative immunofluorescence images of (**A**) IL-22R (red), Lgr5 (green) and (**B**) p-STAT3 (green) in organoids (scale bar, 100 μm). Quantitative analysis of (**C**) Lgr5, (**D**) IL-22R and (**E**) p-STAT3 fluorescence intensity in organoids. (**F**) Western blotting analysis of Lgr5, β-catenin, p-STAT3 and STAT3 in organoids. (**G**) qRT-PCR analysis of IL-22R in organoids. (**H**) ELISA analysis of IL-22 in the culture supernatant of the LPMC -organoid coculture system. Data are expressed as the mean ± SD. * *p* < 0.05, ** *p* < 0.01, **** *p* < 0.0001; NS, no significance. +, with. −, without.

To further explore the mechanism by which L-fucose mediates the growth of ISCs through IL-22, we detected the downstream indicators related to the proliferation of ISCs. IL-22R is a complex formed by two subunits (IL-22RA1 and IL-10RB) that is mainly distributed on nonhematopoietic stem cells, and the IL-22/IL-22R axis plays a key pivotal role between immune function and intestinal barrier function connections [25]. As shown in Figure 7A–E, L-fucose stimulated the budding and area of organoids with increased IL-22R-positive and pSTAT3-positive cells. However, the addition of anti-IL-22 antibody inhibited the stimulatory effect of L-fucose on organoids. Western blot and qRT-PCR analyses further verified the immunofluorescence results (Figure 7F,G). In addition, IL-22 was increased in the culture medium by L-fucose intervention, with a similar trend as above (Figure 7H). These results suggest that L-fucose stimulates the growth of intestinal organoids by IL-22-IL-22R-mediated phosphorylation of STAT3.

### 3.8. L-Fucose Improves the Regeneration of ISCs through IL-22 after TNF-α Treatment

**Figure 8:**
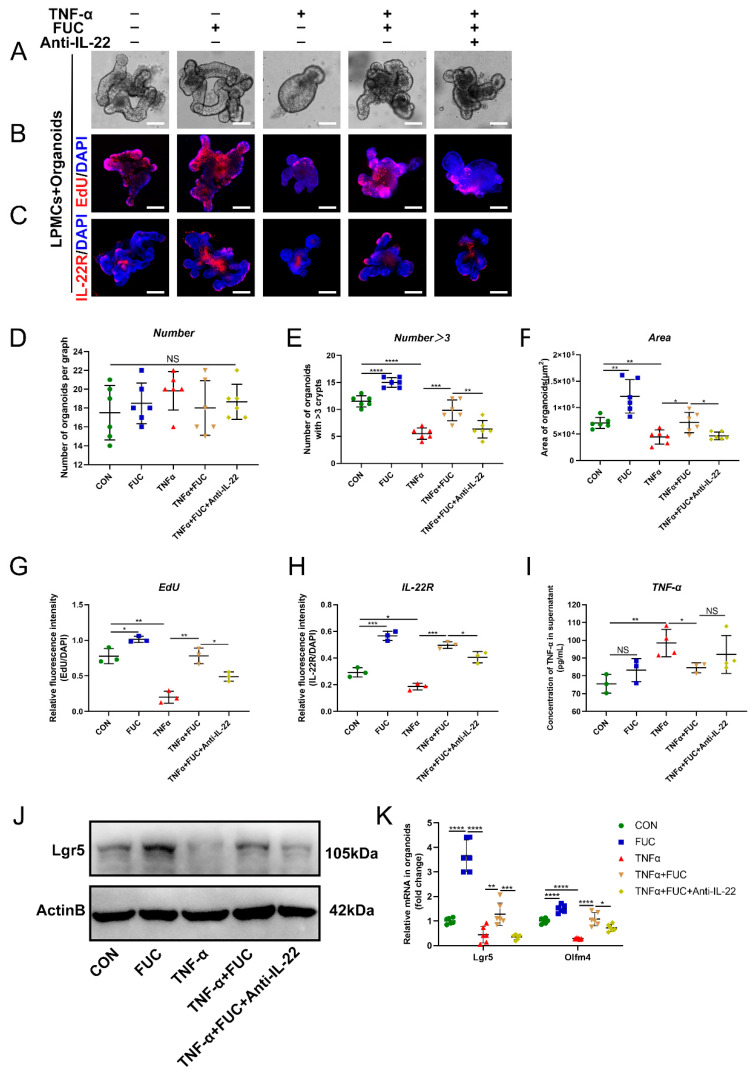
**Restoration of TNF-α-induced ISC damage through IL-22 after L-fucose treatment.** (**A**) Representative images of organoids under a light microscope (scale bar, 100 μm). (**B**) Representative images of EdU (red) staining of organoids (scale bar, 100 μm). (**C**) Representative images of IL-22R (red) immunofluorescence in organoids (scale bar, 100 μm). (**D**) Number of organoids per field of view. (**E**) Number of organoids with more than 3 buds per graph. (**F**) Average area of organoids per graph. Quantitative analysis of (**G**) EdU and (**H**) IL-22R fluorescence intensity in organoids. (**I**) ELISA analysis of TNF-α in the culture supernatant of the LPMC-organoid coculture system. (**J**) Western blotting analysis of Lgr5 protein in organoids. (**K**) qRT-PCR analysis of Lgr5 and Olfm4 in organoids. Data are expressed as the mean ± SD. * *p* < 0.05, ** *p* < 0.01, *** *p* < 0.001, **** *p* < 0.0001; NS, no significance. +, with. −, without.

To demonstrate whether L-fucose promotes ISC regeneration through IL-22, we induced an organoid injury model in the coculture system with TNF-α. ELISA showed that the TNF-α level was significantly increased in the medium of the coculture system after TNF-α induction, indicating the successful establishment of an inflammatory model (Figure 8I). As shown in Figure 8A, the area of organoids was reduced, and the number of buds decreased after TNF-α damage. The administration of L-fucose significantly restored the growth status of organoids, while anti-IL-22 antibody inhibited its growth-promoting effect (Figure 8D–F). Moreover, EdU staining also reflected the same trend of organoid proliferation (Figure 8B,G). Western blot and qRT-PCR analyses of ISC marker genes verified the regeneration of injured organoids by L-fucose treatment (Figure 8J,K). We further examined the expression of IL-22R in organoids and found that TNF-α damaged the level of IL-22R in organoids, while L-fucose increased its expression (Figure 8C,H). These results demonstrate that L-fucose restores injured organoids by IL-22 and IL-22R on ISCs.

## 4. Discussion

**Figure 9:**
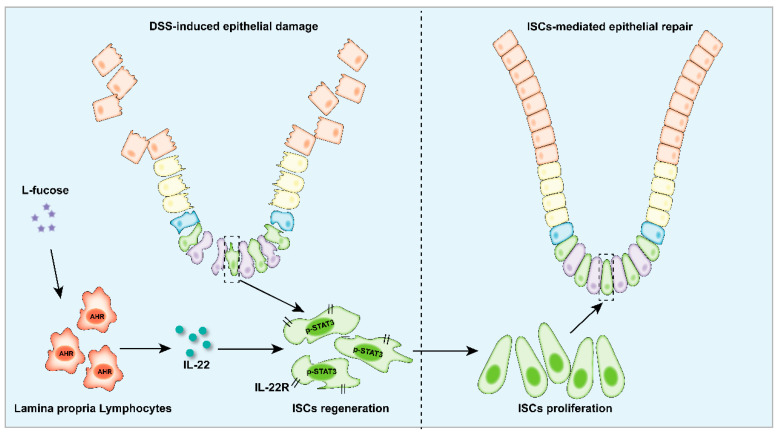
Schematic depiction of the stimulatory effects of L-fucose on LPMCs and ISCs in the colitis colon.

Here, our study demonstrated that L-fucose could alleviate colon inflammation and restore the intestinal epithelial barrier in DSS-induced colitis mice, and it worked through promoting the regeneration and proliferation of ISCs. In addition, in the presence of L-fucose, IL-22 and its related transcription factor AHR were significantly elevated in the colons of colitis mice. In an in vitro experiment, we found that L-fucose stimulated the secretion of IL-22 from CD4^+^ T cells in mouse spleen cells and ILC3 cells in LPMCs through activation of nuclear AHR. Further studies showed that the stimulatory effect of L-fucose on ISC regeneration and proliferation was mediated by IL-22 being released from LPMCs in the LPMC-intestinal organoid coculture experiment. These results indicated that L-fucose indirectly acted on ISCs through LPMCs, thus helping the recovery of damaged intestinal epithelium (Figure 9).

Inflammatory mucosal healing is the primary goal of IBD treatment to improve clinical symptoms, disease recurrence and resection-free survival. ISCs can generate various types of mature intestinal epithelial cells, so the maintenance of their stemness is the key to the continuous renewal and repair of the intestinal mucosal epithelium after injury [21,26]. As a dietary sugar with diverse biological functions, L-fucose has been demonstrated to effectively ameliorate DSS-induced acute and chronic colitis [16,17,18]. In this study, we found that L-fucose significantly relieved colon inflammation and restored the intestinal epithelial barrier, which was probably mediated by ISC proliferation. Our results showed that the mRNA levels of ISC maker genes, including Lgr5, Olfm4 and Ascl2, were significantly increased in the colon of colitis mice after L-fucose treatment. Ki67 and Lgr5 staining in colon tissues revealed rapid cell proliferation at the base of the crypt. Western blot analysis of Lgr5 protein in the colon also supported the stimulatory effect of L-fucose on ISCs.

It is well known that the intestinal immune microenvironment, including innate and adaptive immunity, is related to the maintenance of intestinal homeostasis [27]. With the development of in vitro intestinal organoid culture and single-cell RNA sequencing technology, the connection between ISCs and immune cells is increasingly understood. Recent studies have found that immune cells such as T cells, ILCs, and cytokines secreted by these cells are essential in the regeneration of ISCs and intestinal epithelium [28]. Numerous studies have shown that homeostatic maintenance and postinjury repair of ISCs are IL-22-dependent [29,30,31]. IL-22, mainly produced by group 3 innate lymphoid cells (ILC3s) and CD4^+^ T cells, acts as an important regulator of the DNA damage response in ISCs. Given the important role of IL-22 in the maintenance of ISC homeostasis, we found that the exogenous administration of L-fucose significantly increased intestinal IL-22 secretion and IL-22R expression in DSS-induced chronic colitis. The in vitro experiments also showed increased production of IL-22 from CD4^+^ T cells and ILC3 cells after L-fucose treatment.

The literature has reported that AHR-mediated signaling controls the production of IL-22 [31]. AHR is a ligand-dependent transcription factor and conserved environmental sensor [32] that is linked to a variety of molecular chaperones and remains inactive in the cytoplasm. When the ligand acts on AHR, the AHR-ligand complex moves from the cytoplasm to the nucleus, where it binds to the xeno-response element (XRE), which in turn promotes the transcription of downstream target genes [33]. The activation of AHR can promote the synthesis of IL-22, and AHR can also cooperate with RORγt to induce the secretion of IL-22 in ILC3 cells [34,35]. In addition, many studies have delved into the relationship between AHR and IBD. For example, AHR expression in ILC3s is reduced in inflamed intestinal tissue [36], AHR^−/−^ mice typically exhibit mild spontaneous enteritis [37], DSS-induced enteritis is more severe in AHR^−/−^ mice than in wild-type mice [38], and AHR^−/−^ mice are more susceptible to Citrobacter infection [39]. To explore the pathway by which L-fucose acts on CD4^+^ T cells and LPMCs, we found that the mRNA levels of AHR and its target gene, CYP1A1, were significantly elevated in the colons of colitis mice by L-fucose treatment. The increased nuclear AHR protein in colon cells indicated the activation of AHR signaling. In an in vitro experiment, the AHR inhibitor CH223191 significantly reduced the stimulatory effect of L-fucose on IL-22 secretion from CD4^+^ T cells and LPMCs, which demonstrated that L-fucose promoted IL-22 release through the activation of AHR in CD4^+^ T cells and LPMCs. However, the examination of the role of AHR was solely based on pharmacological inhibition, and the absence of AHR gene deletion in CD4^+^ T cells and LPMCs was a limitation of this study.

IL-22R is widely distributed in the cell membrane of ISCs and is used to receive IL-22 signaling [40]. We found that L-fucose significantly increased the expression of IL-22R at the bottom of organoid crypts in the coculture system, which was attributed to the increased expression of IL-22. Anti-IL-22 antibody inhibited the pro-IL-22R expression effect of L-fucose. STAT3 is a key factor in activating the IL-22 signaling pathway [41]. To verify the activation effect of IL-22 on the downstream signaling of ISCs, we examined the expression of STAT3 and p-STAT3 in organoids. The results showed that the phosphorylation of STAT3 was activated in the coculture system after L-fucose treatment but inhibited by anti-IL-22 antibody intervention. These results confirmed that L-fucose stimulated the proliferation of ISCs through IL-22 secretion by LPMCs via the IL-22R-p-STAT3 pathway.

In summary, our data demonstrated that L-fucose played a therapeutic role in ameliorating DSS-induced chronic colitis in mice by accelerating ISC proliferation, and it works through the AHR/IL-22 pathway of LPMCs. These results prove that L-fucose may be a promising dietetic treatment for IBD. However, further research is needed to verify the specific therapeutic effect and mechanism in clinical trials.

## Supplementary Materials

The following supporting information can be downloaded at: https://www.mdpi.com/article/10.3390/nu14224789/s1, Table S1: Sequences of primers for qRT-PCR analysis; Figure S1: Experiment of different concentrations of L-fucose intervening in coculture system; Figure S2: The effect of L-fucose on intestinal organoids.

## Abbreviations

IBD	inflammatory bowel disease
FUC	L-fucose
ISC	intestinal stem cell
LPMCs	intestinal lamina propria monocytes
EdU	5-ethynyl-2′-deoxyuridine
ILC3	group 3 innate lymphoid cell
DSS	dextran sodium sulfate
DAI	disease activity index
AHR	aromatic hydrocarbon receptors
CYP1A1	cytochrome P450, family 1, subfamily A, polypeptide 1
CH223191	specific antagonists of the aryl hydrocarbon receptor
