# Supplementary material for: Promoting Effect of L-Fucose on the Regeneration of Intestinal Stem Cells through AHR/IL-22 Pathway of Intestinal Lamina Propria Monocytes
Source: Nutrients. 2022 Nov 12;14(22):4789. doi: 10.3390/nu14224789 (PMC9695883; doi:10.3390/nu14224789)
Supplement: Supplementary file 1 [file nutrients-14-04789-s001.zip › nutrients-1990582-SI.pdf]

Supplement Table S1. Sequences of primers for qRT-PCR analysis.

Gene	Forward primer (5′-3′)	Reverse primer (5′-3′)
AHR	TTGGTTGTGATGCCAAAGGGC	CATGCGGATGTGGGATTCTGC
CYP1A1	GGGTTTGACACAGTCACAACT	GGGACGAAGGATGAATGCCG
NOTCH	GCAACTGTCCTCTGCCATATAC	GTCTTCAGACTCCTTGCATACC
Hes	CAACACGACACCGGACAAAC	CGGAGGTGCTTCACAGTCAT
STAT5	GAAGACCAAGTTCATCTGTGTGAC	GGTTTCAGCTCCTCACATGG
IL-22	ATGAGTTTTTCCCTTATGGGGAC	GCTGGAAGTTGGACACCTCAA
GAPDH	CATGGCCTTCCGTGTTCCTA	TACTTGGCAGGTTTCTCCAGG
LGR5	CCTACTCGAAGACTTACCCAGT	GCATTGGGGTGAATGATAGCA
OLFM4	CAGCCACTTTCCAATTTCACTG	GCTGGACATACTCCTTCACCTTA
ASCL2	AAGCACACCTTGACTGGTACG	AAGTGGACGTTTGCACCTTCA

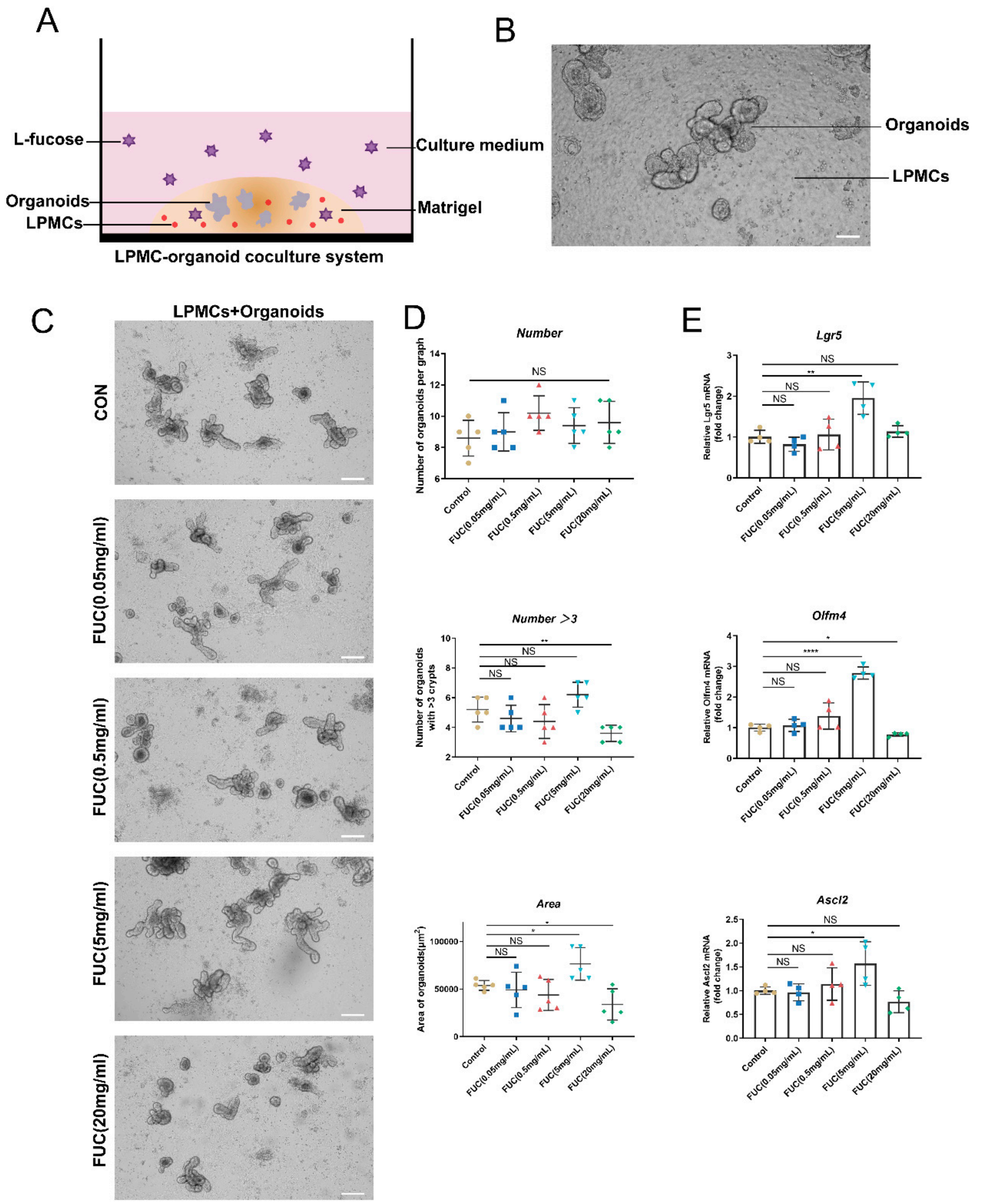

Supplement Figure S1. Experiment of different concentrations of L-fucose intervening in coculture system. (**A**) The coculture model of LPMCs and organoids. (**B**) Organoids cultured with LPMCs were observed with a light microscope (scale bar, 200 μm). (**C**) Different concentrations of L-fucose intervening in co-culture system were observed with a light microscope (scale bar, 200 μm). (**D**) The number of organoids, the number of organoids with more than 3 buds and the average area of organoids per graph. the average area of organoids per graph. (**E**) qRT-PCR analysis of

Lgr5, Olfm4 and Ascl2 in organoids. Data are expressed as the mean ± SD. * $p < 0.05$, ** $p < 0.01$, **** $p < 0.0001$; NS, no significance.

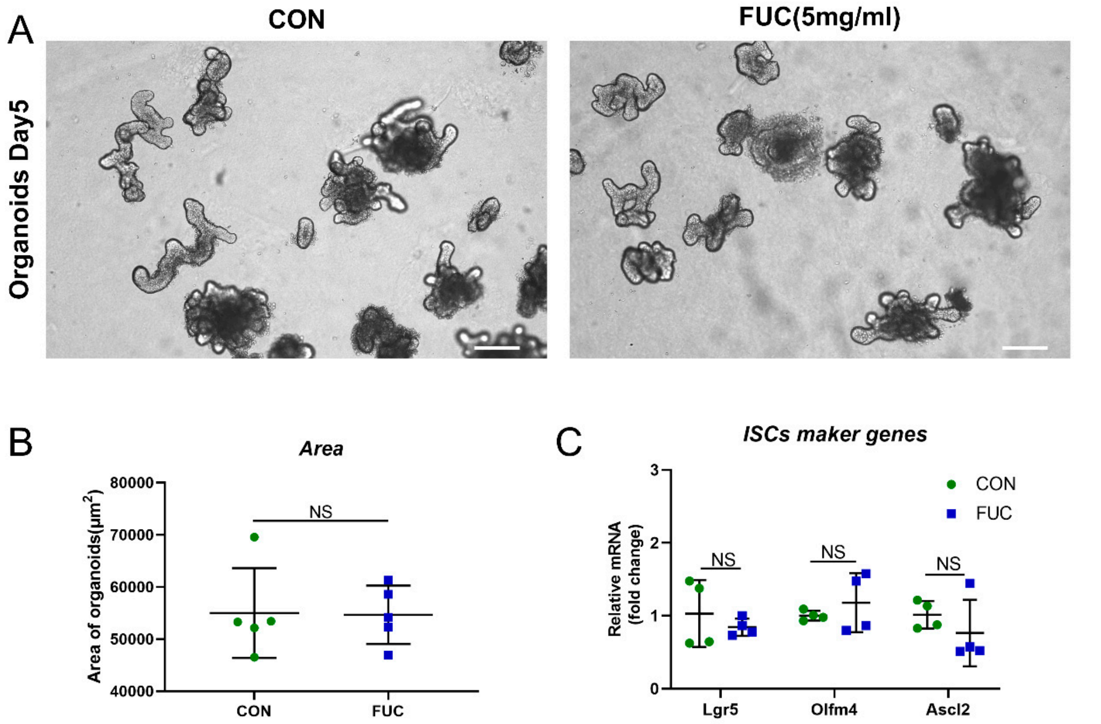

Supplement Figure S2. The effect of L-fucose on intestinal organoids. (**A**) The growth of organoids was observed under a light microscope (scale bar, 200 μm). (**B**) The average area of organoids per graph. (**C**) qRT-PCR analysis of Lgr5, Olfm4 and Ascl2 in organoids. Data are expressed as the mean ± SD. NS, no significance.
